# Supplementary material for: Local heterogeneity of normal lung parenchyma and small airways disease are associated with COPD severity and progression
Source: Respir Res. 2024 Feb 28;25:106. doi: 10.1186/s12931-024-02729-x (PMC10903150; doi:10.1186/s12931-024-02729-x)
Supplement: Supplementary file 1 — Supplementary Material 1 [file 12931_2024_2729_MOESM1_ESM.docx]

Local Heterogeneity of Normal Lung Parenchyma and Small Airways Disease Are Associated with COPD Severity and Progression

Alexander J. Bell, PhD^a^; Ravi Pal, PhD^a^; Wassim W. Labaki, MD, MS^b^; Benjamin A. Hoff, PhD^a^; Jennifer M. Wang, MD^b^; Susan Murray, PhD^c^; Ella A. Kazerooni, MD, MS^a,b^; Stefanie Galban, PhD^a^; David A. Lynch, MB BCh^d^; Stephen M. Humphries, PhD^d^; Fernando J. Martinez, PhD^e^; Charles R. Hatt, PhD^f^; MeiLan K. Han, MD^b^; Sundaresh Ram, MS, PhD^a,g^ (co-last author); Craig J. Galban, PhD^a,g*^

^a^ Department of Radiology, University of Michigan, Ann Arbor, MI, United States

^b^ Department of Internal Medicine, Division of Pulmonary and Critical Care Medicine, University of Michigan, Ann Arbor, MI, United States

^c^ School of Public Health, University of Michigan, Ann Arbor, MI, United States

^d^ Department of Radiology, National Jewish Health, Denver, CO, United States

^e^ Weill Cornell Medical College, New York, NY, United States

^f^ Imbio, LLC, Minneapolis, MN, United States

^g^ Department of Biomedical Engineering, University of Michigan, Ann Arbor, MI, United States

^*^Corresponding author (Email: [cgalban@med.umich.edu](mailto:cgalban@med.umich.edu))

Supplemental Information Guide

| **Item** | **Title/Description** |
| --- | --- |
| Supplemental Methods 1 | Quality Control (QC) Protocol |
| Supplemental Figure 1 | Workflow of Dictionary Learning Model |
| Supplemental Methods 2 | Dictionary Learning Algorithm |
| Supplemental Figure 2 | Boxplots for tPRM Metrics Mean Breadth (B) and Surface Area (S) for all PRM classes, grouped by GOLD Stage |
| Supplemental Table 1 | Multivariable Regression for non-COPD Subset |
| Supplemental Figure 3 | Dictionary Learning Results for a 70 yr old male diagnosed at baseline with GOLD 1 COPD |
| Supplemental Figure 4 | Case Study showing the relationship between V and χ at the local level |

# Supplemental Methods 1: Quality Control (QC) Protocol

QC was performed in two steps (e.g., see 3 and 4 in exclusion diagram below), using GOLD grade, segmented lung volume change ($\Delta$V = inspiration volume – expiration volume, as a function of segmented voxels), and a correlation test metric (Q) defined as the absolute value of the difference between standard scores of FEV_1_% predicted and %PRM^Norm^, reported to be highly correlated in COPD studies (1).


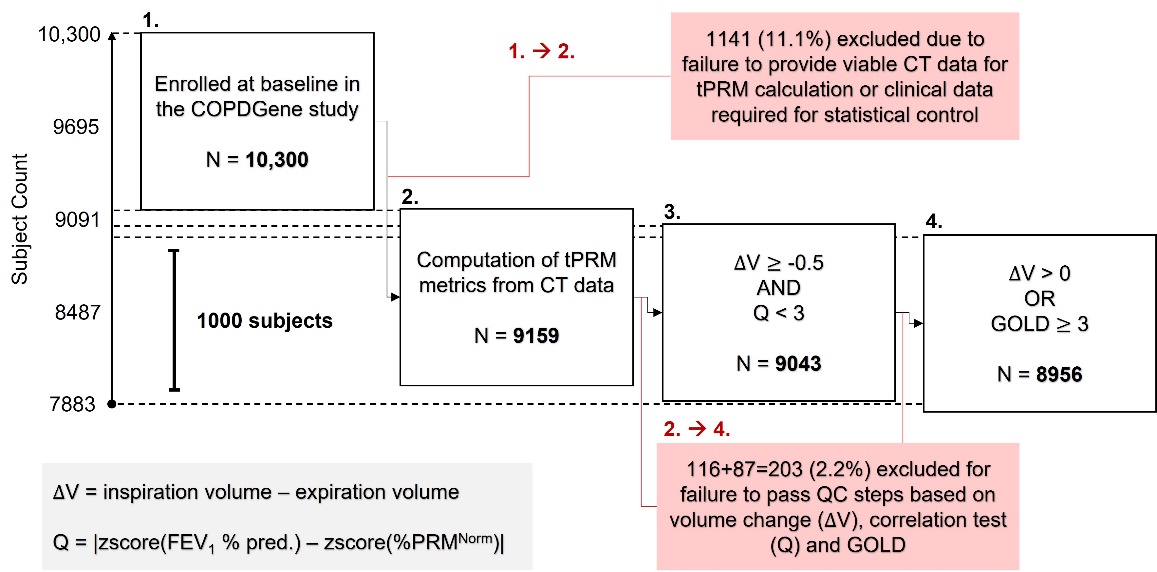


Specifically, imaging QC tests for exclusion were applied consecutively as follows:

1. **∆V < -0.5 L OR Q ≥ 3**

A large negative volume change, here defined as greater than 0.5 L, often indicates transposition of intended respiratory stages (expiration/inspiration), due to faulty maneuver or data handling error. In addition, we test here if a deviation of equal to or greater than 3 standard deviations from the expected positive correlation between FEV_1_% predicted and %PRM^Norm^ has occurred.

1. **∆V ≤ 0 L and GOLD < 3**

This second step goes on to test if a non-severe COPD participant (GOLD < 3) has zero or negative volume change. N.B. here and in step 1 we have considered that there may be participants with severe disease that have some abnormal volume changes (close to 0 due to very limited lung function).

# Supplemental Figure 1: Workflow of Dictionary Learning Model

Class $c_{1}$

Class $c_{2}$


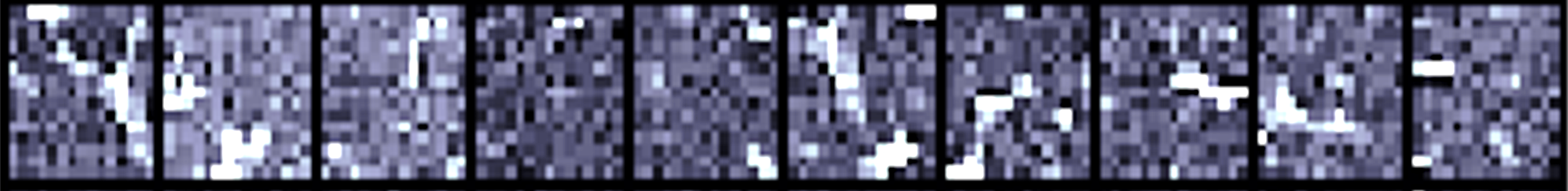

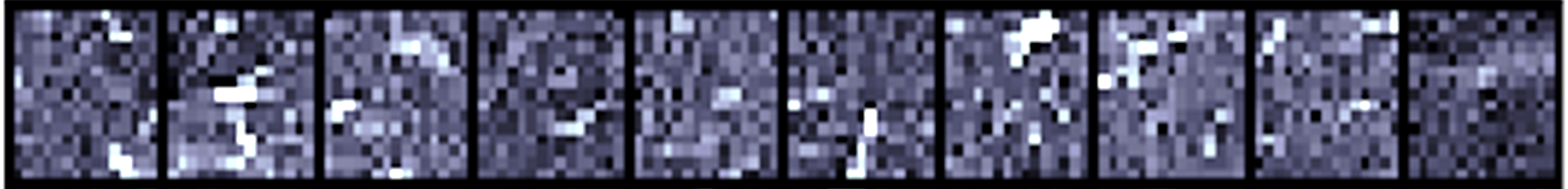

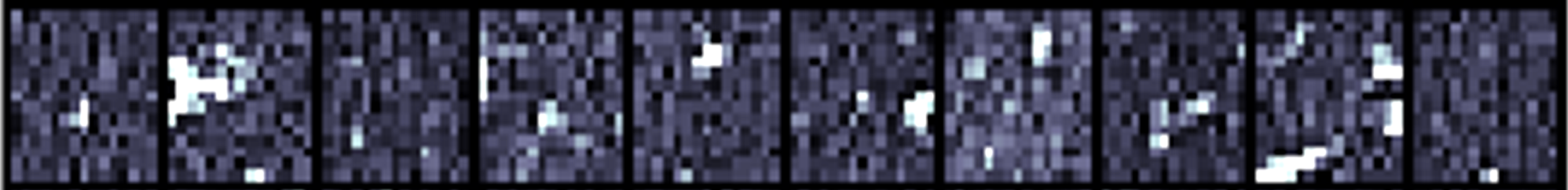

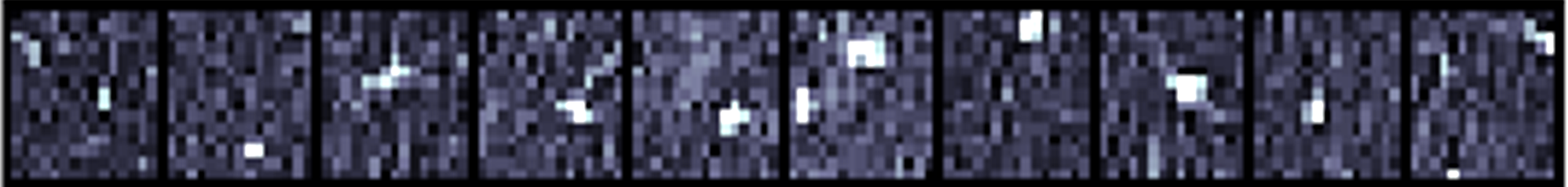

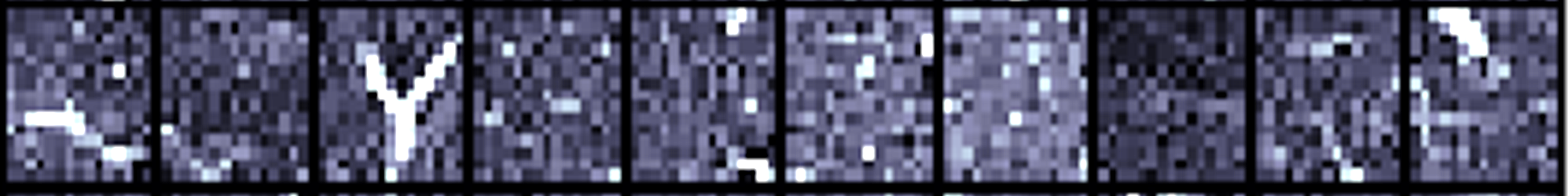

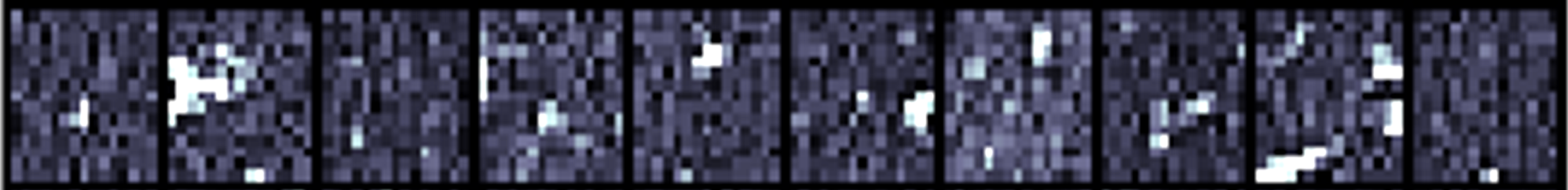

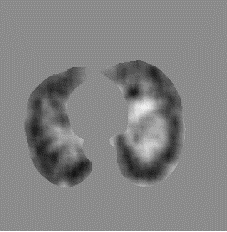

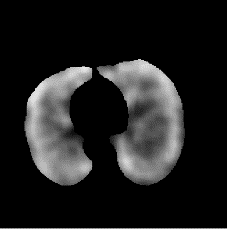

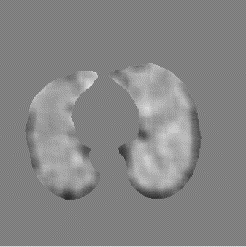

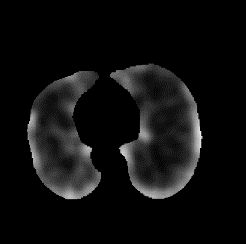

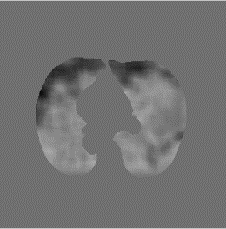

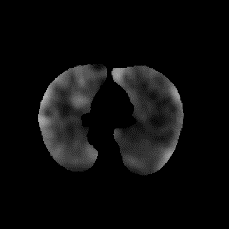

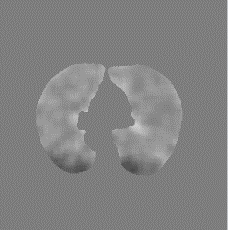

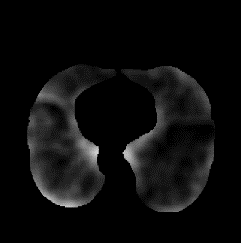


V^fSAD^

X^fSAD^

X^Norm^

V^Norm^

V^Norm^

X^Norm^

V^fSAD^

X^fSAD^


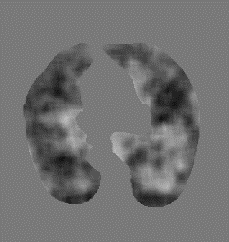

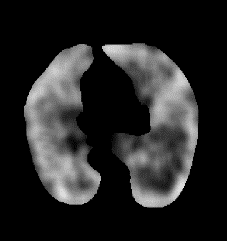

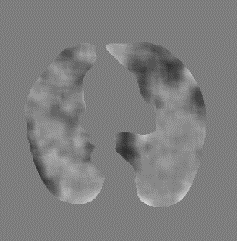

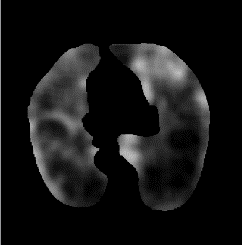

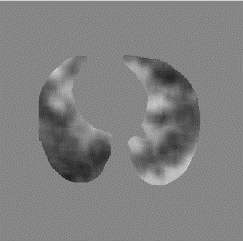

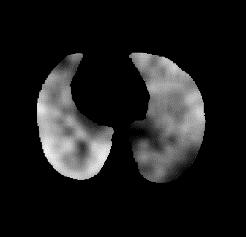

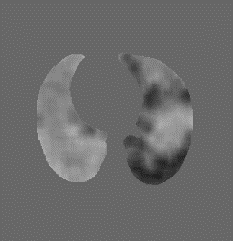

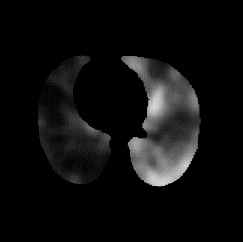


**Step 1: Train D**

**Step 2: Find 𝛉**

**Step 3: Classify**

V^Norm^

X^Norm^

V^fSAD^

X^fSAD^

X^fSAD^

V^Norm^

X^Norm^

V^fSAD^


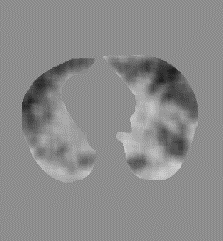

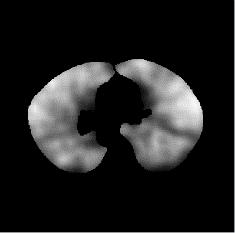

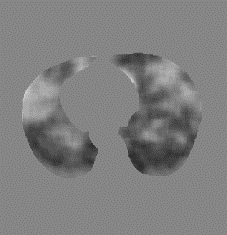

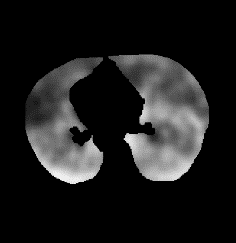


V^Norm^

X^Norm^

V^fSAD^

X^fSAD^


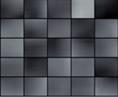

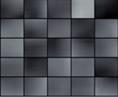

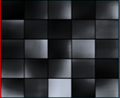

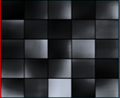


Randomly Extracted 2D Image Patches

Class Specific Dictionary Training

Classification on a set of 2D Patches

Classify on a set of 2D Patches

Class

Label

Labeled 3D Training Images

Labeled 3D Training Images

Test 3D Image

Threshold θ


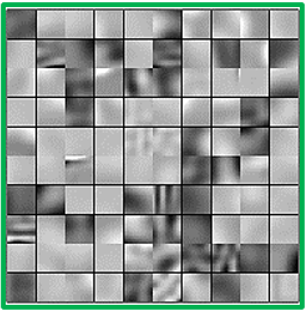

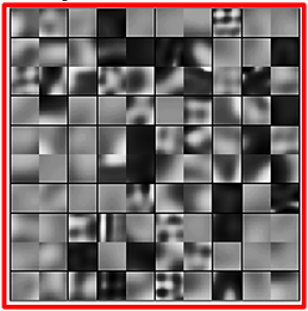


# **Supplemental Figure 1:** Flowchart describing the various steps involved in the proposed dictionary learning algorithm. Step 1: 2D image patches are extracted from each of the prior maps (V^Norm^, V^fSAD^, χ^Norm^, and χ^fSAD^) in the labeled training data and a class specific dictionary is trained for the entire class. Step 2: all 2D patches from each 3D prior image map are classified and a threshold value is selected to classify the entire case as belonging to one of the classes. Step 3: The learned class specific dictionaries and the threshold for the entire case are used to classify the test images.

# Supplemental Methods 2: Dictionary Learning Algorithm

Image sparsity has emerged as a significant property of images and sparsity-based regularization has been used for various image processing applications (2-9). Sparse image representations are at the heart of many modern approaches to medical image classification and include (10-12). The sparse model assumes that each patch within an image can be accurately represented using a few elements of a basis set called a dictionary. For image classification problems a separate class-specific dictionary is learnt from patches belonging to each class of images. In this work, we have developed a multiview task-driven dictionary learning algorithm – a novel approach that aims to learn discriminative dictionaries for each class from multiple views of the data in a joint fashion by imposing group sparsity constraints (13).

***Dictionary Learning*:** Our proposed method utilizes an overcomplete dictionary $\mathcal{D}$ constructed from the CT images, which is an $n \times K$ matrix whose columns represent $K$ “atoms” of size $n,$ where an “atom” is a sparse coefficient vector (i.e., a vector of weights/coefficients in the sparse basis). We train a separate dictionary for each class. Each dictionary $\mathcal{D}_{i}$ represents the image patches from class $i$ reasonably well, but at the same time represents the image patches from the other classes quite poorly. There are several ways to train/learn a dictionary (14). In this work, we have adopted the task-driven dictionary learning algorithm proposed by Mairal et al. (15). To train them, we solve a combinatorial optimization problem, where an approximate solution is obtained by alternating between a greedy sparse coding step using the current dictionary estimate, and a dictionary update step.

***Sparse Coding:*** We assume that any image patch $x$ in a CT image can be represented as a sparse linear combination of the atoms of the dictionary $\mathcal{D}$ as: $x \approx\mathcal{D}\alpha,$ where $\alpha$ is the sparse coefficient vector. Given a dictionary, $\mathcal{D,}$ the goal in sparse coding is to find a sparse coefficient vector $\alpha.$ This requires solving a second optimization problem, the optimal solution to which is found using a greedy approach such as an orthogonal matching pursuit algorithm (16).

***Classification:*** In sparse representation-based classification, an image patch $x$ is classified according to how well the patch is represented by the class-specific dictionaries. Once a dictionary $\mathcal{D}_{i}$ has been trained for each class $i$, classification of a new image patch $x_{\mathrm{new}}$ is performed by evaluating the reconstruction/representation errors for different classes. From the class representation errors a pseudo-probability measure $P_{i}$ is computed and the image patch is assigned to the class that has the maximum probability value.

***Training:*** The dictionary learning model was trained on a desktop workstation running a 64-bit Windows operating system (Windows 10) with an Intel Xeon W-2123 CPU at 3.6GHz with 128GB DDR4 RAM. The x-, y-, and z-dimensions of each image in our dataset were x = 512, y = 512, and z ~ 1250. CT lung scans from N = 4483 cases from the COPDGene phase 1 dataset who had follow up examination were considered. A representative 2D slice of a donor lung CT image is shown in **Figure 1A** of the manuscript. The lungs within these CT images were then automatically segmented using in-house software developed using MATLAB R2020a (MathWorks, Natick, MA). Our dataset for this study consists of a total N = 4483 lung CT images belonging to two categories: i) N = 1516 CT lungs that were described as fast progressors with a change of FEV_1_ ≥ -60ml/yr, referred to as class 1, and ii) N = 2967 CT lungs that were described as slow progressors with a change of < -60ml/yr in their FEV_1_, referred to as class 2. We used 35% of the data for training and the remaining 65% for testing. A total of 8,000,000 2D image patches from the three (axial, coronal, and sagittal) views from each of the prior maps (tPRM maps V^Norm^, V^fSAD^, χ^Norm^, and χ^fSAD^) were extracted from the training data for each class to train the dictionaries. The proposed dictionary learning algorithm was developed using MATLAB R2020a software (MathWorks, Natick, MA). We used the sparse modeling software (SPAMS) toolbox (17) for the orthogonal matching pursuit optimization algorithm to efficiently optimize the dictionary elements. The hyper parameters of the dictionary learning algorithm include the image patch size $l,$ the number of dictionary bases $K$ for each dictionary, the sparsity controlling parameter $\lambda,$ and the positive regularization parameter $\rho$ in sparse coding. The optimal values for these parameters were automatically selected on a validation set (randomly chosen from within the training data) using the receiver operating characteristic (ROC) curves, by varying one parameter at a time while keeping the others fixed and choosing that value of the parameter that maximizes the area under the curve (AUC) of the ROC curve. The parameters of the dictionary learning algorithm were set to $l=25,$ $K=512,$ $\lambda=0.0015,$ and $\rho=0.006.$

# Supplemental Figure 2: Boxplots for tPRM Metrics Mean Breadth (B) and Surface Area (S) for all PRM classes, grouped by GOLD Stage

Mean breadth (**B**)

**A)**


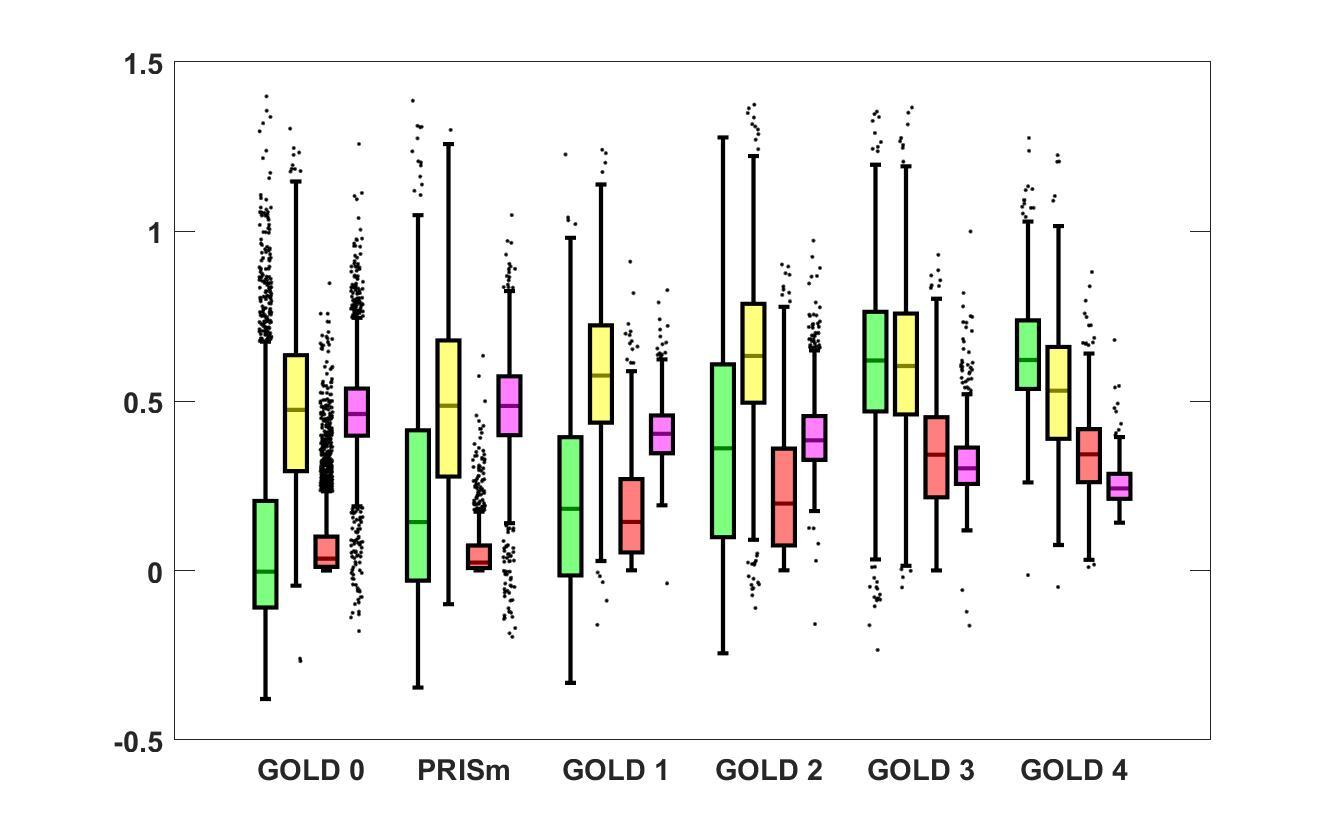


Surface area (**S**)

**B)**


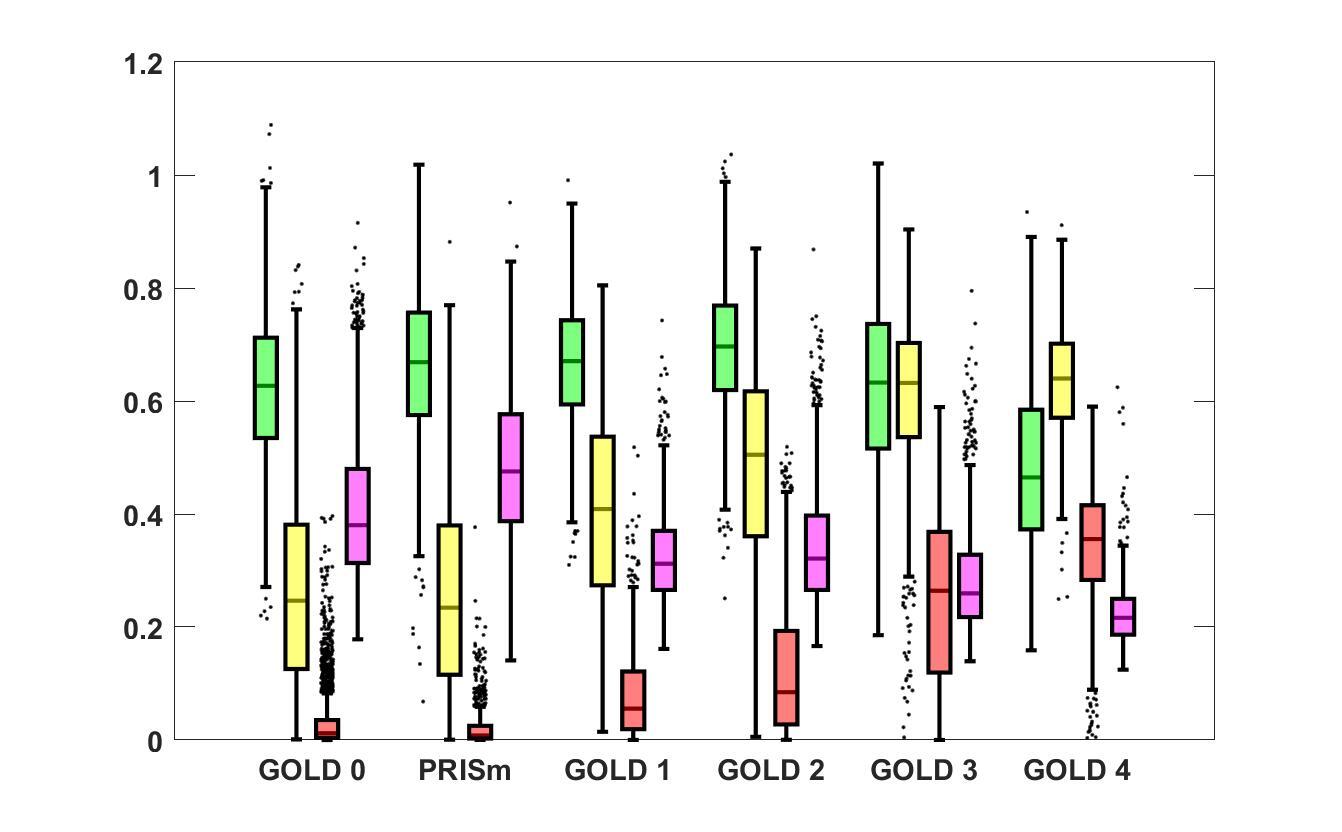


**Norm**

**fSAD**

**Emph**

**PD**

outlier

# **Supplemental Figure 2:** Boxplots for topological measures of PRM maps PRM^Norm^ (green), PRM^fSAD^ (yellow), PRM^Emph^ (red) and PRM^PD^ (magenta) across all GOLD stages, “at-risk” (GOLD 0), and PRISm. Plots of (A) surface area, describing class exposure (exposed faces of voxels). B) mean breadth, which can be interpreted as average curvature (convex/concave) of class surfaces. Box plots were computed following standard protocol for box and whiskers; box lines determined by lower quartile (Q1), middle quartile / median (Q2) and upper quartile (Q3), and whiskers are drawn out to Q1 – 1.5 x IQR and Q3 + 1.5 x IQR for lower and upper limits, respectively. IQR = Q3-Q1. Outliers are defined as points beyond the given upper and lower limits and illustrated as black points with a random bounded horizontal perturbation beyond box whiskers.

# Supplemental Table 1: Multivariable Regression for non-COPD Subset

| Performance | FEV_1_pp | FEV_1_/FVC | FEF_25-75_ (L) | V^Emph^ |
| --- | --- | --- | --- | --- |
| Adjusted R^2^ | 0.143 | 0.126 | 0.265 | 0.263 |
| SE | 14.39 | 0.048 | 0.883 | 0.014 |
| Age (yrs) |  | -0.119** | -0.290** | 0.042* |
| Sex (M/F) | -0.027 (0.05) | 0.014 (0.298) | -0.381** | -0.062** |
| BMI (kg/cm^2^) | -0.075** | 0.104** | 0.116** | -0.130** |
| Smoking (Pack-Years) | -0.105** | -0.098** | -0.098** |  |
| CT vendor | -0.052** |  | -0.015 (0.252) | 0.185** |
| Race |  | 0.117** | -0.068** |  |
| V^Norm^ | 0.325** | -0.044* | 0.109** | -0.157** |
| V^fSAD^ | 0.134** | -0.218** | -0.075** | 0.373** |
| χ^Norm^ |  |  |  | -0.038 (0.01) |
| χ^fSAD^ | -0.105** | -0.075** | -0.058** |  |

**Notes:** Multivariable regression modelling using volume density (V) and Euler-Poincaré Characteristic (χ) for PRM-derived Normal and fSAD (introduced stepwise) to model pulmonary function test measures in the COPD subset. Each column presents results for a different regression model. FEV_1_pp, forced expiratory volume in one second percent predicted; FEV_1_, forced expiratory volume in one second; FVC, forced vital capacity; FEF_25-75_, forced mid-expiratory flow; Emph, emphysema; SE, standard error of the estimate; BMI, body mass index; Norm, normal; and fSAD, functional small airways disease. Model performance is reported as adjusted R^2^ and standard error of the estimate. Feature association is reported as standardized beta coefficients (β); cells for stepwise variables removed from final model. All regression models were controlled for age, sex, race, BMI, pack years and CT vendor. P values ≥ 0.01, < 0.01 and ≥ 0.001, and < 0.001 are presented as values in parentheses, *, and **, respectively.

# Supplemental Figure 3: Dictionary Learning Results on a 70 yr old male diagnosed at baseline with GOLD 1 COPD.

**Exp. CT Scan**

**V^fSAD^ = 0.115**

**Patch Probability Maps**


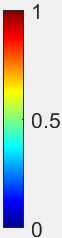

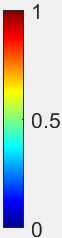


1

0

1


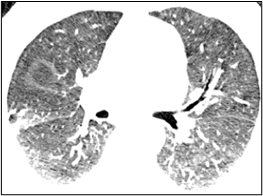

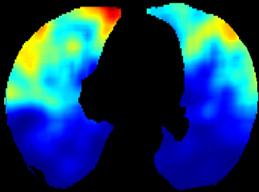

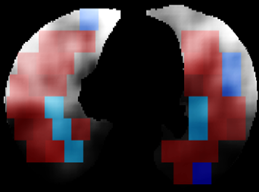


Normal


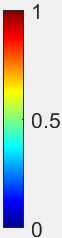


0.5

**PRM**

0.03

**χ^fSAD^ = 0.015**

-0.03


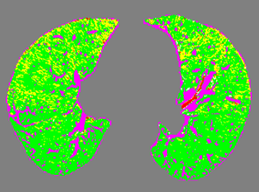

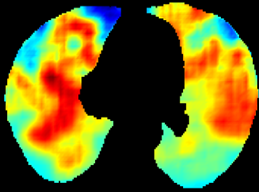

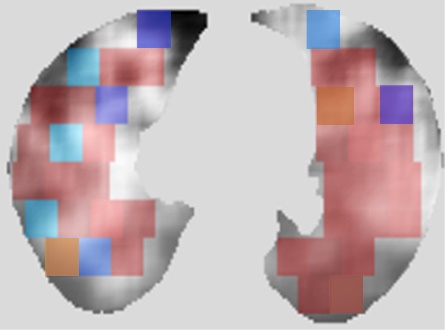


Abnormal

0

**Supplemental Figure 3**: The dictionary learning results for a 70 yr old male diagnosed at baseline with GOLD 1 COPD and declared a slow progressor with ΔFEV_1_/yr of 101 ml/yr. This case was correctly identified by our ML algorithm as a slow progressor. Representative axial slice of an expiratory CT scan, its associated PRM map, the tPRM maps V^fSAD^ and χ^fSAD^ of PRM^fSAD^, and their image patch probability maps from the dictionary learning model.

# Supplemental Figure 4: Case Study showing the relationship between V and χ at the local level.

B


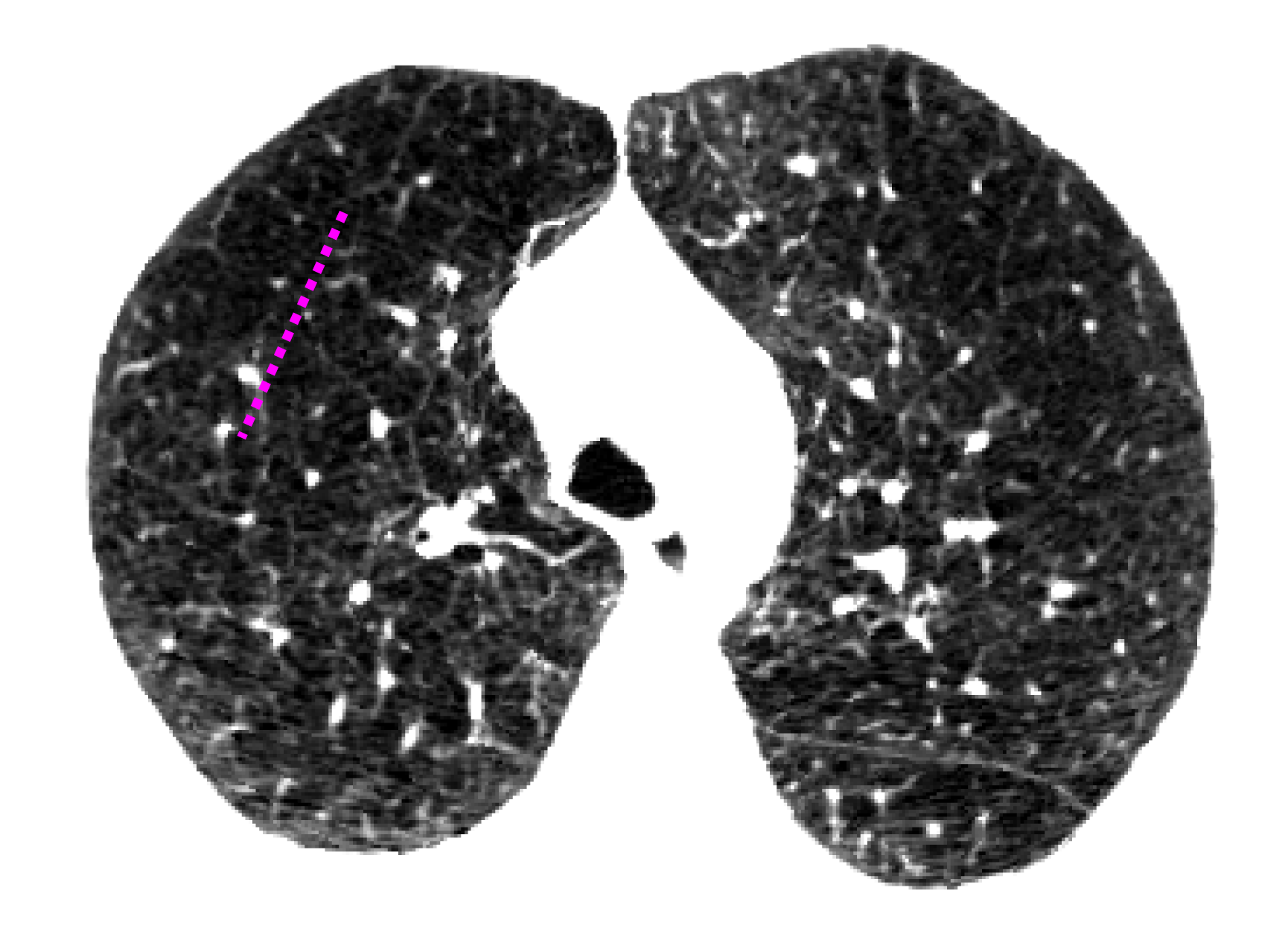


A


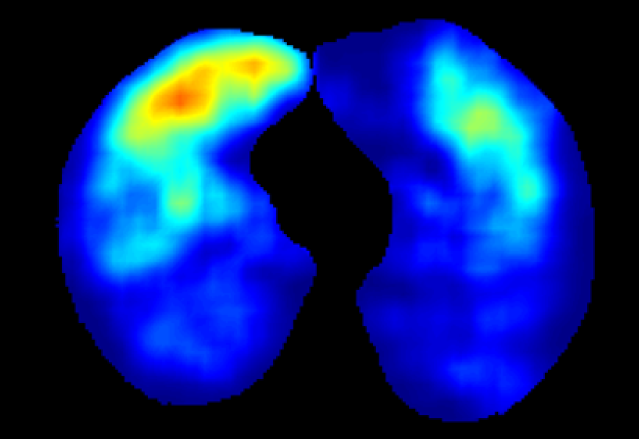

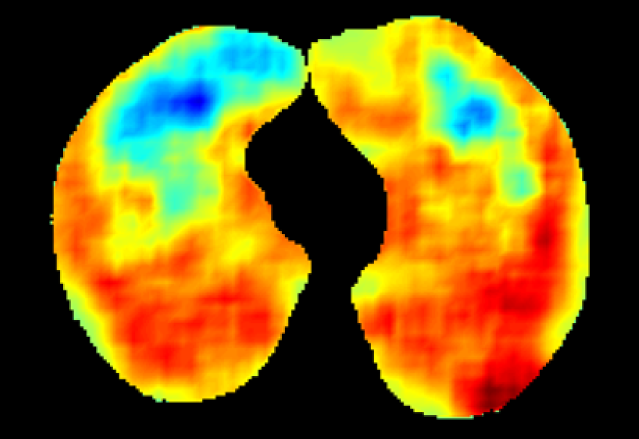


PRM^Emph^

V

χ


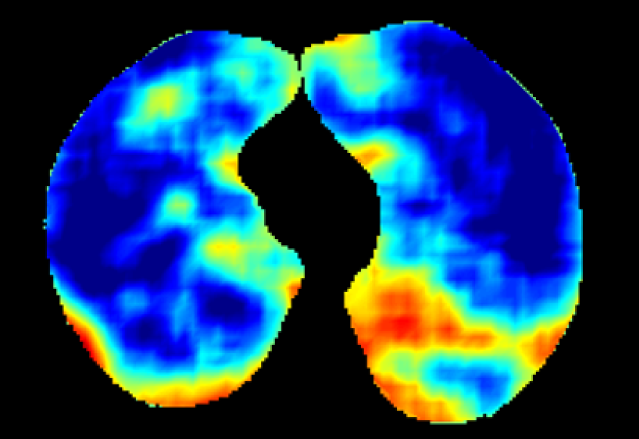

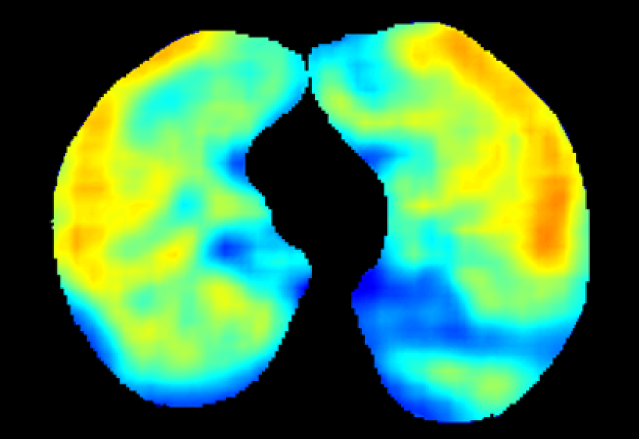


PRM^fSAD^

χ

V

C


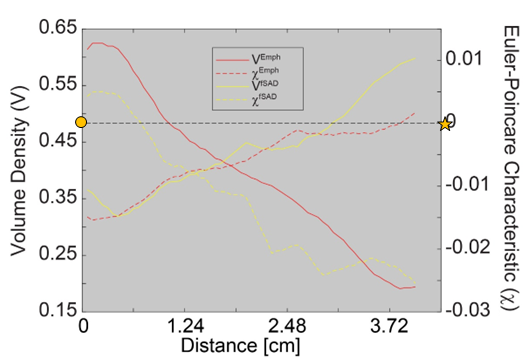


**Supplemental Figure 4:** Case study demonstrating the spatial relationship between the topologies of PRM^fSAD^ and PRM^Emph^. The case is a female subject, 48 years of age, diagnosed with GOLD 4 COPD. Single axial slice from (A) spatially aligned CT scan acquired at full inflation with corresponding (B) slices from V and χ of PRM^fSAD^ and PRM^Emph^. (C) Topology values were plotted along the dashed line on the CT slice, starting from circle to star. Lines on plot were color coded to match PRM classification (red signifies PRM^Emph^ and yellow signifies PRM^fSAD^). Solid and dashed lines indicate V (left y-axis) and χ (right y-axis).

References

1. Galbán CJ, Han MK, Boes JL, Chughtai KA, Meyer CR, Johnson TD, et al. Computed tomography–based biomarker provides unique signature for diagnosis of COPD phenotypes and disease progression. Nat Med. 2012;18(11):1711-5.

2. Ding D, Ram S, Rodriguez JJ. Image Inpainting Using Nonlocal Texture Matching and Nonlinear Filtering. IEEE Trans Image Process. 2019;28(4):1705-19.

3. Malladi SRSP, Ram S, Rodríguez JJ. Image Denoising Using Superpixel-Based PCA. IEEE Transactions on Multimedia. 2021;23:2297-309.

4. Ram S, Rodríguez JJ, editors. Single image super-resolution using dictionary-based local regression. 2014 Southwest Symposium on Image Analysis and Interpretation; 2014 6-8 April 2014.

5. Ram S, Rodriguez JJ, editors. Image super-resolution using graph regularized block sparse representation. 2016 IEEE Southwest Symposium on Image Analysis and Interpretation (SSIAI); 2016 6-8 March 2016.

6. Ram S. Sparse Representations and Nonlinear Image Processing for Inverse Imaging Solutions: The University of Arizona; 2017.

7. Ram S, Hoff BA, Bell AJ, Galban S, Fortuna AB, Weinheimer O, et al. Improved detection of air trapping on expiratory computed tomography using deep learning. PLoS One. 2021;16(3):e0248902.

8. Ram S, Tang W, Bell AJ, Pal R, Spencer C, Buschhaus A, et al. Lung cancer lesion detection in histopathology images using graph-based sparse PCA network. Neoplasia. 2023;42:100911.

9. Ding D. Image Inpainting Based on Exemplars and Sparse Representation: The University of Arizona; 2017.

10. Sarkar R, Acton ST. SDL: Saliency-Based Dictionary Learning Framework for Image Similarity. IEEE Trans Image Process. 2018;27(2):749-63.

11. Zhang S, Zhan Y, Metaxas DN. Deformable segmentation via sparse representation and dictionary learning. Med Image Anal. 2012;16(7):1385-96.

12. Diamant I, Klang E, Amitai M, Konen E, Goldberger J, Greenspan H. Task-Driven Dictionary Learning Based on Mutual Information for Medical Image Classification. IEEE Trans Biomed Eng. 2017;64(6):1380-92.

13. Ram S, Verleden SE, Kumar M, Bell AJ, Pal R, Ordies S, et al. Computed tomography-based machine learning for donor lung screening before transplantation. J Heart Lung Transplant. 2023.

14. Rubinstein R, Bruckstein AM, Elad M. Dictionaries for Sparse Representation Modeling. Proceedings of the IEEE. 2010;98(6):1045-57.

15. Mairal J, Bach F, Ponce J. Task-Driven Dictionary Learning. IEEE Transactions on Pattern Analysis and Machine Intelligence. 2012;34(4):791-804.

16. Tropp JA, Gilbert AC. Signal Recovery From Random Measurements Via Orthogonal Matching Pursuit. IEEE Transactions on Information Theory. 2007;53(12):4655-66.

17. Mairal J, Bach F, Ponce J. Sparse modeling for image and vision processing. arXiv preprint arXiv:14113230. 2014.
